# Interprofessional collaborative care characteristics and the occurrence of bedside interprofessional rounds: a cross-sectional analysis

**DOI:** 10.1186/s12913-016-1714-x

**Published:** 2016-09-01

**Authors:** Jed D. Gonzalo, Judy Himes, Brian McGillen, Vicki Shifflet, Erik Lehman

**Affiliations:** 1 Medicine and Public Health Sciences, Health Systems Education, Pennsylvania State University College of Medicine, Hershey, PA USA; 2 Nursing Medical Services, Neuroscience, and Cancer Institute, Penn State Hershey Medical Center, Hershey, PA USA; 3 General Medicine Acute Care Unit, Penn State Hershey Medical Center, Hershey, PA USA; 4 Public Health Sciences, Pennsylvania State University College of Medicine, Hershey, PA USA; 5 Division of General Internal Medicine, Penn State Hershey Medical Center – HO34, 500 University Drive, Hershey, PA 17033 USA

**Keywords:** Interprofessional collaborative care, Relational coordination, Team-based care, Health services research, Patient-centered care, Hospital-based medicine, Quality improvement

## Abstract

**Background:**

Interprofessional collaboration improves the quality of medical care, but integration into inpatient workflow has been limited. Identification of systems-based factors promoting or diminishing bedside interprofessional rounds (BIR), one method of interprofessional collaboration, is critical for potential improvements in collaboration in hospital settings. The objective of this study was to determine whether the percentage of bedside interprofessional rounds in 18 hospital-based clinical units is attributable to spatial, staffing, patient, or nursing perception characteristics.

**Methods:**

A prospective, cross-sectional assessment of data obtained from nursing audits in one large academic medical center on a sampling of hospitalized pediatric and adult patients in 18 units from November 2012 to October 2013 was performed. The primary outcome was the percentage of bedside interprofessional rounds, defined as encounters including one attending-level physician and a nurse discussing the case at the patient’s bedside. Logistic regression models were constructed with four covariate domains: (1) spatial characteristics (unit type, bed number, square feet per bed), (2) staffing characteristics (nurse-to-patient ratios, admitting services to unit), (3) patient-level characteristics (length of stay, severity of illness), and (4) nursing perceptions of collegiality, staffing, and use of rounding scripts.

**Results:**

Of 29,173 patients assessed during 1241 audited unit-days, 21,493 patients received BIR (74 %, range 35-97 %). Factors independently associated with increased occurrence of bedside interprofessional rounds were: intensive care unit (odds ratio 9.63, [CI 5.30-17.42]), intermediate care unit (odds ratio 2.84, [CI 1.37-5.87]), hospital length of stay 5-7 days (odds ratio 1.89, [CI, 1.05-3.38]) and >7 days (odds ratio 2.27, [CI, 1.28-4.02]), use of rounding script (odds ratio 2.20, [CI 1.15-4.23]), and perceived provider/leadership support (odds ratio 3.25, [CI 1.83-5.77]).

**Conclusions:**

Variation of bedside interprofessional rounds was more attributable to unit type and perceived support rather than spatial or relationship characteristics amongst providers. Strategies for transforming the value of hospital care may require a reconfiguration of care delivery toward more integrated practice units.

**Electronic supplementary material:**

The online version of this article (doi:10.1186/s12913-016-1714-x) contains supplementary material, which is available to authorized users.

## Background

Interprofessional collaborative care (IPCC) is the process through which different professional groups work together to improve healthcare quality [1–4]. Providers of different professions working as a team promotes improved communication, coordination of care, and patient-centered shared-decision making [5, 6]. Given the emerging evidence of the positive impact of IPCC on outcomes, work processes integrating IPCC models into healthcare delivery is a national health policy focus specifically in the proposed changes in the Affordable Care Act [1, 2, 7, 8]. Although there is a need to accelerate and transform healthcare delivery to be more team-based and patient centered, implementation of IPCC methods in hospital-based units has not been well studied [9].

Factors promoting care coordination and teamwork in hospital-based units include routines, such as treatment pathways, individuals serving boundary-spanning roles, and team meetings [10]. Hospitalized patients’ care involves mutual relationships, collaboration, and decision-making between all healthcare providers and patients, highlighting the need for IPCC methods to improve quality [1]. Bedside interprofessional rounds (BIR) including both physicians and nursing staff are a primary method of promoting collaboration in hospital-based settings [4, 11–13]. However, studies investigating the occurrence of BIR in medicine, pediatrics, and intensive care units demonstrate a wide variation in frequency from 1-80 % [14–17]. To our knowledge, no studies have investigated the incidence of BIR across different hospital-based units, or identified unit-level collaboration-related characteristics associated with BIR. Identification of systems-based factors promoting or diminishing the frequency of BIR is vital for providing potential improvement targets for this patient-centered activity.

Starting in 2012, our institution introduced a new quality metric related to BIR, defined as nurses and physicians working together at the bedside during rounds. In this study, we sought to: (1) examine the percentage of patients receiving BIR in 18 different units within our hospital, and, (2) determine whether the percentage of BIR is attributable to four categories of variables, including spatial, staffing, patient, and nursing perception characteristics. We hypothesized intensive care unit settings, higher nurse-to-patient ratios, and smaller unit sizes would be associated with a higher percentage of BIR.

## Methods

### Study design

Following a hospital-wide initiative to increase BIR, from November 2012-October 2013, we performed a prospective cross-sectional assessment of data obtained from nursing audits completed during ≥5 days per month in 18 hospital units. The Institutional Review Board determined this study did not meet the definition of human subjects research and therefore more formal submission and approval was not required.

### Study setting

The study was conducted at a 501-bed university-based acute care hospital in central Pennsylvania. Our hospital provides a full spectrum of medical and surgical care for pediatric and adult patients. In 2012, our hospital leadership sought to improve IPCC between providers and patients. The primary expectation was for all frontline teams to perform BIR on ≥80 % of patients per day in each unit. To obtain mutual understanding amongst providers and set clear expectations for continual assessment, an *a priori* definition was established for BIR: “*encounters that include at least one attending-level physician (from the primary team) and nurse discussing the case at the patient’s bedside*.”

### Study outcomes

The primary outcome was the percentage of BIR occurring in each unit. For the covariates, since the literature has not identified specific categories of system or collaboration-related factors associated with BIR, we undertook an exploratory approach to variable selection. Through research team meetings, informal interviews, a literature review, and our work on medicine-based BIR, we developed four categories of variables hypothesized to affect BIR (Tables 1 and 2) [18, 19]. First, to address the spatial-related factors that may promote IPCC, we selected several variables, including unit type (acute, intermediate, intensive care), number of beds in unit, and square feet in unit per bed. Staffing and service factors included nurse-to-patient ratios and number of admitting services in unit per bed, calculated by dividing the number of different admitting services admitting ≥5 patients to the unit during the study period by number of unit beds. This variable was developed to reflect the degree of team variability in each unit. Patient characteristics included hospital length-of-stay for patients admitted to each unit, and severity of illness measured by the APR-DRG, a variable derived from billing data [20]. Nursing perceptions of nurse-physician collegiality, staffing adequacy, provider support, and use of a BIR script were evaluated.

**Table 1:** Characteristics of hospital-based units (*n* = 18) in the Penn State Hershey Medical Center

Unit	Spatial Characteristics			Staffing/Service		Patient Characteristics		Nursing Perceptions			
	Unit Type ^a^	No. of Beds	Sq. Ft per bed	Nurse-patient ratio	Admitting Services per bed^b^	Length of Stay	Severity of Illness^c^	Collegiality^d^	Staffing^d^	Rounding Script^e^	Support Score^f^
Pediatric Intensive Care	3	18	878	1:1.5	0.22	8.95	2.83	2.99	1.95	7	21
Neonatal Intensive Care	3	31	303	1:2	0.03	25.27	2.79	2.49	2.66	5	17
Surgical Intensive Care	3	30	553	1:2	0.63	7.96	2.98	2.48	2.33	7	19
Medical Intensive Care	3	16	597	1:2	0.38	8.62	3.33	2.95	2.90	4	18
Neurology	1,2,3	35	672	1:2.5	0.17	5.75	2.47	3.11	2.73	3	16
Heart and Vascular Cardiac Care	3	15	666	1:2	0.47	7.84	2.73	2.87	3.00	2	13
Cancer Institute	1,2	39	435	1:4	0.33	5.15	2.28	2.91	2.64	3	17
Heart and Vascular Progressive Care	1,2	24	398	1:3.5	0.25	5.56	2.37	2.84	3.12	5	18
Pediatric Hematology-Oncology Service	1	16	987	1:2.5	0.38	6.18	2.19	3.15	2.88	6	17
Women’s Health	1	24	203	1:4.5	0.17	6.10	1.42	3.30	2.68	6	21
Pediatric Intermediate Care	2	17	872	1:2.5	0.59	3.97	2.20	3.01	2.53	4	15
Pediatric Acute Care	1	36	411	1:3.5	0.28	3.10	1.84	2.99	2.72	5	18
Medical Intermediate Care	2	20	470	1:3	0.30	7.09	2.74	2.81	2.42	1	16
General Surgery	1	18	496	1:4.5	0.61	3.54	2.11	3.10	2.54	3	19
Internal/Family Medicine	1	44	385	1:4	0.20	4.45	2.50	3.17	2.77	2	17
General Surgery/Neurology	1	44	385	1:4.5	0.34	4.02	2.21	2.87	2.48	1	12
General Surgery	1	42	404	1:4.5	0.50	4.60	2.36	2.78	2.55	4	16
Flex/Observation	1	14	765	1:4.5	0.93	5.01	2.44	-	-	3	16

^a^ Unit Type: 3 = intensive care, 2 = intermediate care, 1 = general acute

^b^ Number of different services admitting ≥5 patients to unit in one-year period/number of unit beds

^c^ Derived from billing data (APR-DRG value)

^d^ Scores obtained from Collegial Nurse-Physician Relations/Staffing/Resource Adequacy domain from Practice Environment of the Nursing Work Index; flex/observation had a “float” pool of nurses, thereby could not receive a survey; responses 4 = strongly agree, 3 = agree, 2 = disagree, 1 = strongly disagree

^e^ Reported by units’ nursing leadership on a 1-7 scale (1 = not at all, 7 = a great extent)

^f^ Summation score from 3 domains on a 1-7 scale (1 = not at all, 7 = a great extent), max score 21

**Table 2:** Frequency of patients receiving bedside interprofessional rounds by unit (*n* = 18) at the Penn State Hershey Medical Center (Nov. 2012-Dec. 2013)

Unit	No. of Days ^a^	Total Patients	Ave. census/day	No. of Patients Receiving BIR	Frequency of BIR
Pediatric Intensive Care	63	755	11.98	733	0.97
Neonatal Intensive Care	59	1812	30.71	1732	0.96
Surgical Intensive Care	66	1622	24.58	1546	0.95
Medical Intensive Care	72	1091	15.15	1003	0.92
Neurology	72	2192	30.44	1784	0.81
Heart and Vascular Cardiac Care	66	1806	27.36	1465	0.81
Cancer Institute	69	2380	34.49	1917	0.81
Heart and Vascular Progressive Care	69	1623	23.52	1294	0.80
Pediatric Hematology-Oncology Service	69	1066	15.45	844	0.79
Women’s Health	71	1569	22.10	1224	0.78
Pediatric Intermediate Care	80	1148	14.35	861	0.75
Pediatric Acute Care	77	1358	17.64	1015	0.75
Medical Intermediate Care	70	1175	16.79	862	0.73
General Surgery	71	925	13.03	653	0.71
Internal/Family Medicine	71	3065	43.17	2004	0.65
General Surgery/Neurology	63	2553	40.52	1214	0.48
General Surgery	67	2708	40.42	1227	0.45
Flex/Observation	66	325	4.92	115	0.35

^a^ Number of days during the study when audits performed

### Data sources and collection

To monitor the success of the hospital-wide BIR initiative, each unit’s nurse manager/charge nurse performed “audits” on ≥5 randomly selected days each month during the 12-month period. The nursing-audit process involved asking each bedside nurse to report how many of his/her patients received BIR according to the definition on that day. At month’s end, each unit submitted tallies to the Department of Nursing, which were posted on the hospital’s Quality Dashboard.

Covariates were obtained from several sources. For spatial characteristics, we obtained and analyzed the floor plans for each unit. For patient- and service-level characteristics, we used our hospital’s clinical data warehouse to acquire the number of admitting services to the unit per bed, length-of-stay, and severity of illness. For nursing perceptions of nurse-physician relations and staffing adequacy, we used scores from the National Database of Nursing Quality Indicators Practice Environment Scale of the Nursing Work Index (PES-NWI) in the domain of Collegial Nurse-Physician Relations (three items) and Staffing/Resource Adequacy (four items) obtained during the study period (Appendix 1). The “flex/observation” unit was not included in the PES-NWI survey because nurses were from a float pool originating from several units. For nurse-to-patient ratios, perceived support, and use of a BIR script, we administered a paper-based survey in May 2014 to each unit’s nurse manager. Questions related to unit characteristics and included quantitative and Likert-scale questions (Additional file 1).

### Data analysis

Descriptive statistics were used to report characteristics of each unit, patient census, and BIR frequency. The primary outcome (percentage of BIR) was calculated as the sum of all patients receiving BIR divided by the sum of the unit’s census from all recorded audits for each day and multiplied by 100 %. Percent BIR was not normally distributed and was difficult to analyze with parametric analysis. Therefore, we stratified percent BIR into two groups based around the median: high (≥80 %) and low (<80 %) to keep the bias low; based on prior studies, this cut off also served as an ideal and reasonable target for BIR [4, 21]. Because our binary outcome variable was measured repeatedly over time within each unit, a generalized estimating equations (GEE) model was used to identify predictors of the main outcome. Odds ratios were used to quantify the magnitude and direction of significant associations. A multivariable GEE model with all significant predictors (*p* < 0.05) from bivariate analysis was used to determine if each predictor maintained its significance when adjusted for the others. A check for multicollinearity between predictor variables was made using variance inflation factors (VIF) statistics from linear regression prior to applying the multivariable model. All VIF statistics for variables included in the model were below 5. The final reduced model fit for the significant predictor variables was checked against the starting full model that included all predictor variables using QIC statistics for GEE model comparison, and the QIC was higher in the model including only the significant predictor variables. Data were analyzed using SAS 9.4 (Cary, NC).

## Results

### Characteristics of units and collaboration factors

Of 18 units, six were intensive-care units (ICU), four were intermediate care units, and eight were acute care units (Table 1). Average number of beds per unit was 27 (range 14-44), with a mean 549 square feet per bed (range 203-987). Nurse-to-patient ratio mean was 1 to 3.2 (range 1.5-4.5). Patients’ mean length-of-stay was 6.8 days (range 3.1-25.3) and severity of illness was 2.43 (range 1.4-3.3).

### Bedside interprofessional rounds

During the study period, 29,173 patients (mean 23.5 patients per unit per day) were assessed during 1241 audited unit-days, with 21,493 patients receiving BIR (74 %, range 35-97 %, Table 2).

### Factors associated with bedside interprofessional rounds

Factors independently associated with increased occurrence of BIR were intensive care unit (OR 9.63, [CI 5.30-17.42], vs. acute-care), intermediate care unit (OR 2.84, [CI 1.37-5.87], vs. acute-care), length-of-stay 5-7 days (OR 1.89, [CI, 1.05-3.38], vs. <5 days) and >7 days (OR 2.27, [CI, 1.28-4.02], vs. <5 days), use of rounding script (OR 2.20, [CI 1.15-4.23], score ≥ 4 vs. <4), and perceived provider/leadership support (OR 3.25, [CI 1.83-5.77], score ≥17 vs. <17, Table 3). Number of beds, square feet in unit and per bed, admitting services per bed, nurse-patient ratios, nurse-physician collegiality score, and severity of illness showed no associations with BIR.

**Table 3:** Associations between spatial, staffing, patient, and nursing perception variables and frequency of bedside interprofessional rounds in 18 hospital-based units (total *n* = 1241)

Variable - n (%)	Bedside Interprofessional Rounds, ≥80 % (*n* = 669)	Unadjusted OR (95 % CI)	Adjusted OR (95 % CI)
Spatial Characteristics			
Unit type:			
General care	172 (25.7)	1	1
Intermediate care	158 (23.6)	2.83 (1.19-6.73)	2.84 (1.37-5.87)
Intensive care	339 (50.7)	13.65 (4.30-43.34)	9.63 (5.30-17.42)
Number of unit beds:			
< 19	285 (42.6)	1	
19-35	278 (41.6)	1.57 (0.50-4.88)	
> 35	106 (15.8)	0.30 (0.07-1.19)	
Square feet per bed:			
< 410	160 (23.9)	1	
410-600	268 (40.1)	2.54 (0.61-10.57)	
> 600	241 (36.0)	2.05 (0.53-8.01)	
Staffing/Service			
Nurse-patient ratio:			
> 1:3	228 (34.1)	1	1
≤ 1:3	441 (65.9)	4.71 (1.73-12.85)	1.14 (0.64-2.03)
Number of admitting services in unit/bed:^a^			
< 0.35	370 (55.3)	1	
≥ 0.35	299 (44.7)	0.96 (0.32-2.86)	
Weekday			
No	143 (21.4)	1	
Yes	526 (78.6)	1.19 (0.94-1.51)	
Patient Characteristics			
Hospital length of stay for patients admitted to unit:			
< 5 days	114 (17.0)	1	1
5-7 days	235 (35.1)	3.75 (1.53-9.21)	1.89 (1.05-3.38)
> 7 days	320 (47.8)	12.82 (3.25-50.52)	2.27 (1.28-4.02)
Severity of illness (APR-DRG):			
< 2.4	285 (42.6)	1	
≥ 2.4	384 (57.4)	2.27 (0.75-6.82)	
Nursing Perceptions			
Nurse-physician collegial score:^b^			
< 2.95	302 (46.1)	1	
≥ 2.95	353 (53.9)	0.92 (0.29-2.90)	
Staffing and resource adequacy:^b^			
< 2.67	337 (51.5)	1	
≥ 2.67	318 (48.6)	1.00 (0.33-3.02)	
BIR script score:^c^			
< 4	214 (32.0)	1	1
≥ 4	455 (68.0)	3.18 (1.11-9.13)	2.20 (1.15-4.23)
BIR support score:^d^			
< 17	182 (27.2)	1	1
≥ 17	487 (72.8)	3.24 (1.17-8.97)	3.25 (1.83-5.77)

^a^ Number of different services admitting ≥5 patients to unit in a one-year period/unit beds

^b^ Scores obtained from Collegial Nurse-Physician Relations/Staffing/Resource Adequacy from Practice Environment of the Nursing Work Index (PES-NWI); responses 4 = strongly agree, 3 = agree, 2 = disagree, 1 = strongly disagree

^c^ Reported by units’ nursing leadership on a 1-7 scale (1 = not at all, 7 = a great extent)

^d^ Summation score from 3 domains on a 1-7 scale (1 = not at all, 7 = a great extent), max score 21

^e^ Adjusted for other significant variables in Table 3

## Discussion

In our hospital-based units during the one-year study period, frequency of BIR exceeded 70 %, with higher frequencies occurring in ICUs than intermediate or acute-care units. Additional factors associated with BIR were longer length-of-stay for patients admitted to the unit, and nursing leaderships’ perceived support by providers and use of a BIR script; besides unit type, spatial characteristics were not associated with BIR. These results advance our understanding about factors impacting the occurrence of BIR in hospital units, and highlight potential barriers hindering ideal patient-centered care for all admitted patients. Awareness of benefits for IPCC is increasing, and potentially will become more integrated into quality performance measures. As a result, IPCC may become more widely used in models of reimbursement for hospitals [8, 22]. Therefore, formal investigations into IPCC processes are required to inform improvement, and offer a theoretical model for informing the redesign of more integrated, hospital-based units achieving higher value.

In considering these results, two issues are critical to the discussion of BIR. First, in the context of “rounds,” IPCC occurring at the bedside is relatively new to the literature. The traditional method of “bedside rounds,” or physician teams rounding at the bedside, has been identified as a patient-centered method for education and care delivery [21, 23–25]. Numerous studies have investigated physician-based bedside rounds in several specialties, including pediatrics, internal medicine, and surgery [14, 26]. These studies highlight that bedside rounds occur at an incidence of <50 % of all encounters, and <20 % of total rounding time [16, 17, 27–29]. However, integration of nurses or healthcare professionals with physicians at the bedside is less studied. Structured interdisciplinary bedside rounds (BIR using a script) and multidisciplinary rounds (interprofessional rounds in a conference room) are two forms of interprofessional rounds, however these concepts either have not been evaluated or do not occur at the bedside, respectively [30–32]. As described in our prior work on the medicine service, BIR occurred in two-thirds of patients, with higher frequencies occurring in intermediate care units (vs. acute-care), with more senior residents and less experienced attending physicians, during weekdays, and lower team census sizes [4]. This study expands on these concepts by assessing BIR not only in one unit or service line but rather in numerous hospital-based units, which, to our knowledge, has not previously been described.

Second, regardless of the type of rounds, the focus in the literature is on the service-line spanning several units rather than the clinical unit providing care for patients assigned to multiple service lines. Although some ICUs may be closed units, most hospital-based units care for patients assigned to a blend of service lines (e.g. medicine, surgical subspecialties) [33]. Individual units have nurses caring for patients admitted to the unit, however other provider groups encompass a highly variable array of physicians, mid-level providers, and allied health professionals. Most of these providers divide their patient care across several units. This provider “migration” creates challenges for optimal patient-centered IPCC, as each unit has different providers, processes, and culture [18, 34]. Prior research on IPCC suggests a general dichotomy between nurses, who tend to be “collectivist” and systems-driven, as compared to physicians who are more “individualists” and autonomy-driven, a schism that may be exacerbated by physician migration between units [35]. Complexities of these systems-, provider-, and team-based factors must be recognized by hospital leadership, providers, and researchers to allow focused consideration into and identification of unit-level (and not only provider-team) factors promoting or diminishing BIR.

Bedside interprofessional rounds occurred far more frequently in ICUs, suggesting characteristics of these units are conducive to BIR. Past work has identified that in medicine service lines, more intermediate-care unit patients receive BIR compared to acute-care units, and physician-based team rounds encounter challenges with geographic dispersion of patients in different units [4, 19, 36]. Our prior work raised the question of the potential frequency of patients who receive BIR, suggesting that for medicine-based units, the maximum is <70 % [4]. Similarly, these results suggest that in units with higher patient-to-nurse ratios and number of admitting services per bed, a significant proportion of patients do not receive BIR.

Collectively, these findings raise the question of how hospital-based units can optimize value by achieving best outcomes at lower costs. With the wide variation of BIR in our hospital, inasmuch as these results are generalizable to other hospitals, the value transformation for hospitalized patients may require a change in the way providers are organized to deliver care [37]. As proposed by Porter and Lee in their work regarding strategies to promote the value transformation, the reorganization of care delivery into Integrated Practice Units (IPUs) potentially can allow frontline providers to collaborate towards a common end and coordinate care most efficiently [37, 38]. Aligning inpatient units toward IPUs, as our results suggest, initially may require an increase in closed units and geographic co-localization of patients [39]. Without such changes, core principles of team-base healthcare delivery and relational coordination, including shared goals, clear roles, and trust, are limited by fragmentation in current processes [10, 40, 41]. These are just two of several potential factors that may promote patient-centered BIR, and high-leverage areas for systems redesign. Patient co-localization by service and provider groups would require extensive changes (e.g. maintaining high census numbers, efficient emergency department throughput) that may prove difficult to achieve [33].

Investigations of collaboration factors promoting optimal work have been performed in management, business and sociology, but less in healthcare [42–45]. Collaboration theory has identified the determinants of successful collaboration within healthcare settings, which includes systemic factors, the social, cultural, and educational systems, interactional determinants and interpersonal relationships, and notably, organizational determinants [46–48]. These organizational determinants include organizational structure, administrative support, team resources, and coordination mechanisms, and suggest factors such as space and policies ensuring team-based meetings to enhance communication promote group processes necessary for collaboration and high levels of teamwork [45, 46, 49]. For example, Prescott and Bowen identified that smaller units may be more conducive to nurse-physician relationships as these groups of providers are closer in physical proximity, thereby promoting collaboration [48]. Top qualities of workplace settings impacting team performance are the workplace’s ability to support distraction-free individual work, impromptu interactions, and informal and formal encounters [45, 50]. Based on these data and our prior experience providing care in hospital-based settings, we hypothesized units with greater square footage and bed number (inverse), and nursing-perceived collegiality and staffing/resource adequacy scores (direct) would be related to BIR, we found no association. The reason for these findings may be that variables were not sensitive enough to detect appropriate associations. In addition to available educational efforts to promote hospital-based IPCC, the identification of variables associated with IPCC are required to guide improvement efforts [3]. Ultimately, the development of a reliable tool to assess a unit’s optimal balance of collaboration characteristics is critical for quality improvement efforts to optimize workflow, coordination, and IPCC in these clinical microsystems [51]. Spatial setting features, their effect on face-to-face interaction and collaborative care processes, and robust assessments of interprofessional collaboration content and quality are necessary in subsequent research to inform the proposed collaboration instrument.

There are several limitations to our study. First, data were obtained from a subset of patients during the study period, and these patients may have had a different case-mix index compared to patients cared for in the unit during unmeasured days, which potentially limits the accuracy of the results. However, this near-time data collection method, also used in our prior work, was resource intensive and diminishes several biases inherent in remote recall [52, 53]. Second, since our institutional goals were related to BIR benchmarks, nursing audits were susceptible to social desirability bias, potentially overestimating BIR frequency. Next, this study was only performed at one academic medical center, limiting the generalizability to other settings, particularly community or non-academic hospitals. Due to technical limitations, several variables were limited in scope. For example, although we used hospital length-of-stay for patients initially admitted to the unit, we could not accurately capture the patient’s length-of-stay within individual units, which is likely a more sensitive variable for IPCC. Given the service-line specificity of Hospital Consumer Assessment of Healthcare Providers and Systems surveys and the mixed nature of our units, we were unable to accurately investigate this relationship. Lastly, these data are from 2012-2013, and given rapid changes to care processes in hospital settings, these findings may be less applicable to current-day settings.

## Conclusions

In this study, BIR frequency was highly variable, ranging from 35-97 %. Factors predicting increased occurrence of BIR were ICUs, hospital length-of-stay for patients admitted to the unit, and nursing leaderships’ perceived support by providers and use of a BIR script. These findings highlight both non-modifiable and modifiable collaboration variables to optimize patient-centered, IPCC in hospital-based units. Future efforts will need to address additional variables associated with this model of IPCC.

## Appendix 1

2013 National Database of Nursing Quality Indicators (NDNQI) RN Survey with Practice Environment Scale©

Description: The Nursing Quality Indicators used in this study, including “staffing and resource adequacy” and “collegial nurse-physician relations,” are shown in Appendix 1.

For each item, please indicate the extent to which you agree that the item is PRESENT IN YOUR CURRENT JOB (all response options: strongly agree, agree, disagree, strongly disagree).

### Staffing and resource adequacy

1. Adequate support services allow me to spend time with my patients
2. Enough time and opportunity to discuss patient care problems with other nurses
3. Enough registered nurses to provide quality patient care.
4. Enough staff to get the work done.

### Collegial nurse-physician relations

1. Physicians and nurses have good working relationships
2. A lot of team work between nurses and physicians.
3. Collaboration (joint practice) between nurses and physicians.

## Additional file

Additional file 1: Nursing Leadership Survey - Bedside Interprofessional Rounds (RN-MD rounding). The Nursing Leadership Survey items used in this study, including characteristics of the nursing unit and perceptions of nurse leadership regarding bedside nurse-physician rounds, are shown in Additional file 1. (DOCX 32 kb)
