# Supplementary material for: Interprofessional collaborative care characteristics and the occurrence of bedside interprofessional rounds: a cross-sectional analysis
Source: BMC Health Serv Res. 2016 Sep 1;16(1):459. doi: 10.1186/s12913-016-1714-x (PMC5007992; doi:10.1186/s12913-016-1714-x)
Supplement: Additional file 1: — Nursing Leadership Survey - Bedside Interprofessional Rounds (RN-MD rounding). The Nursing Leadership Survey items used in this study, including characteristics of the nursing unit and perceptions of nurse leadership regarding bedside nurse-physician rounds, are shown in Additional file 1. (DOCX 32 kb) [file 12913_2016_1714_MOESM1_ESM.docx]

**Additional file 1:**

**Nursing Leadership Survey - Bedside Interprofessional Rounds (RN-MD rounding)**

1. What is your name? _______________
2. Please list the unit(s) you supervise: _______________
3. Please input a response for each of the following items related to your unit:
   1. Average daily census: _____
   2. Number of nurses assigned on your unit per shift:
      1. 7a-3p _____ 3p-11p _____ 11p-7a _____
   3. Average nurse-to-patient ratio per day: _____
   4. Number of beds on your unit: _____
4. The following items had the same Likert-scale response options:
   1. Do the nurses have a script/defined contribution to bedside Nurse/MD Rounds:
   2. In reference to bedside nurse-physician “rounds,” please rate the culture of support for bedside nurse-physician rounds from the nursing staff:
   3. In reference to bedside nurse-physician “rounds,” please rate the culture of support for bedside nurse-physician rounds from the physicians:
   4. In reference to bedside nurse-physician “rounds,” please rate the culture of support for bedside nurse-physician rounds from leadership:

ANSWER CHOICES:

Not at all To a great extent

1 2 3 4 5 6 7
